# Draft genome of *Lactobacillus amylovorus* KSAU with probiotic potential isolated from the gastrointestinal tract of industrial pigs

**DOI:** 10.1128/mra.00577-24

**Published:** 2025-05-21

**Authors:** Yuri A. Lysenko, Alexander V. Milovanov, Sergey V. Kopyltsov, Dominika Gahurová, Anastasia V. Elisiutikova

**Affiliations:** 1 Department of Veterinary Medicine, Russian State Agrarian University Moscow Timiryazev Agricultural Academy 222434 , Moscow, Russia; 2 Department of Botany, Faculty of Natural Sciences, Comenius University in Bratislava 37864 , Bratislava, Slovak Republic; 3 Department of Biotechnology, Biochemistry and Biophysics, Kuban State Agrarian University named after IT Trubilin 64863 , Krasnodar, Krasnodar Krai, Russia; 4 Department of Plant and Animal Genetics, Kuban State Agrarian University named after IT Trubilin 64863 , Krasnodar, Krasnodar Krai, Russia; Wellesley College Department of Biological Sciences, Wellesley, Massachusetts, USA

**Keywords:** *Lactobacillus amylovorus*, gastrointestinal tract, bacteriocins, intestinal cecum

## Abstract

The *Lactobacillus amylovorus* KSAU strain was isolated from the chyme of the intestinal cecum of an industrial pig. Genes encoding the synthesis of bacteriocins were annotated and suggest their potential to improve the preparation and production of probiotics for pigs.

## ANNOUNCEMENT

A current trend in the animal husbandry is the use of probiotic preparations based on lactic acid microorganisms. They suppress pathogens and preserve the beneficial microorganisms in the gastrointestinal tract of farm animals (1). Lactobacillus strains were isolated from the chyme of industrial pigs (provided by the “Piatachok” company) and demonstrated antagonistic properties against *Staphylococcus aureus* and *Escherichia coli*. Isolates were cultivated on the “Bifidum” medium (FBSIS State Research Center for Applied Microbiology and Biotechnology, Obolensk) at 37°C for 48 hours. The isolate with the strongest antagonistic activity was selected for further whole-genome sequencing (WGS) (1).

A liquid bacterial culture was incubated in MRS medium at 38°C for 48 hours. Bacterial culture (0.5 mL) was centrifuged at 13,000 rpm for 5 minutes. DNA was isolated using the DNeasy Blood & Tissue Kit (QIAGEN, Germany). The library for WGS was prepared using the DNA Preparation (M) Tagmentation Kit (Illumina Inc, USA), and the quality and quantity were estimated using a Qubit fluorometer (Thermo Fisher, USA). Sequencing was performed on the MiSeq device, utilizing 2 × 150 bp MiSeq Reagent Micro Kit v2 (Illumina Inc, USA).

Read quality control was performed with FastQC v.0.11.9 (2). Adapters were removed by Trimmomatic v.0.39 (3). *De novo* assembly of the genome was performed using the UGENE program (4) with built-in SPAdes v.3.15.3 (5). The quality of the genome assembly was assessed using QUAST v.5.0.2 (6). Coding sequences (CDs) were annotated with the NCBI Prokaryotic Genome Annotation Pipeline (PGAP) version 6.7 (7). Default parameters were used except where otherwise noted.

A total of 8,923,418 reads were obtained, and the assembly of the *L. amylovorus* KSAU genome resulted in 336 contigs with a final length of 2,194,806 bp, N50 value 33.1 kb, GC content 38%, genome coverage 133.9×, and 2,216 protein-coding sequences. Additionally, 5 rRNAs, 63 tRNAs, 3 noncoding RNAs, 131 pseudogenes, and 11 CDs for synthesis of bacteriocins, including lactococcin and helveticin, were annotated by PGAP. The taxonomic affiliation of the isolate was determined by the digital DNA–DNA hybridization (8) using the Type (Strain) Genome Server (9). The highest percentage of agreement (69.5%) was with the *L. amylovorus* strain DSM 20531 (GCA_002706375.1).

## Data Availability

The genome was deposited in GenBank under the following accession numbers: BioSample SAMN33879542, BioProject PRJNA948185, SRA SRR28111351 and SRR23952874, and GenBank accession number GCA_029477185.2.
